# Measuring Conceptual Associations *via* the Development of the Chinese Visual Remote Associates Test

**DOI:** 10.3389/fpsyg.2022.799928

**Published:** 2022-03-22

**Authors:** Ching-Lin Wu, Pei-Zhen Chen, Hsueh-Chih Chen

**Affiliations:** ^1^Program of Learning Sciences, National Taiwan Normal University, Taipei City, Taiwan; ^2^Institute for Research Excellence in Learning Sciences, National Taiwan Normal University, Taipei City, Taiwan; ^3^Department of Educational Psychology and Counseling, National Taiwan Normal University, Taipei City, Taiwan; ^4^Chinese Language and Technology Center, National Taiwan Normal University, Taipei City, Taiwan

**Keywords:** creativity, picture naming, remote associates test, visualization, free association

## Abstract

Multiple versions of the Chinese Remote Associates Test (CRAT) have been developed. Thus far, all CRATs have employed verbal stimuli; other forms of stimuli have not yet been used. In this context, the present study compiled a Chinese Visual Remote Associates Test (CVRAT) that conforms to the Chinese language and culture based on a picture naming database. The developed CVRAT has two versions, CVRAT-A and CVRAT-B, each comprising 20 test questions. A typical CVRAT question consists of three stimuli pictures, requiring respondents to propose a target word that is semantically associated with all the pictures. When compiling the CVRAT, this study first selected target words, sifted through stimuli words and corresponding pictures, and analyzed pilot test questions. After compilation, their reliability and validity were examined. The results showed that the CVRAT had moderate internal consistency reliability, good criterion-related validity for the Chinese Word Remote Associates Test (CWRAT), Chinese Radical Remote Associates Test (CRRAT), Chinese Compound Remote Associates Test (CCRAT), insight problem-solving, as well as acceptable discriminant validity for fluency, flexibility, and originality of a divergent thinking test. In other words, CVRAT can effectively measure remote associative capability and provides a figural creativity test that facilitates the understanding of different kinds of remote associations.

## Introduction

As a tool widely used to measure creativity, the Remote Associates Test (RAT; Mednick, 1968; Wu et al., 2020) consists of test questions that comprise three, seemingly irrelevant stimuli. It requires respondents to propose a word that is associated with all three stimuli. For example, in an RAT question comprising stimuli words, “fish,” “mine,” and “rush,” one possible solution is “gold,” as the word “gold” can be paired with the stimuli to create the new expressions “goldfish,” “gold mine,” and “gold rush,” respectively. Those who perform well on RAT tend to demonstrate a great ability to create new products with seemingly irrelevant elements, also known as remote associative ability (Mednick, 1962).

Several Chinese RATs (i.e., CRATs) have been developed in recent years (Jen et al., 2004). Different types of verbal CRATs have been compiled, including the Chinese Word Remote Associates Test (CWRAT; Huang et al., 2012), Chinese Radical Remote Associates Test (CRRAT; Chang et al., 2016), and Chinese Compound Remote Associates Test (CCRAT; Wu and Chen, 2017). Unlike the verbal RAT that uses text as a stimulus, visual RAT (i.e., VRAT) has also been investigated (Olteteanu and Falomir, 2015; Toivainen et al., 2019; Olteţeanu and Zunjani, 2020). A VRAT question consists of stimuli pictures which require respondents to identify the pictures and propose a word related to the three pictures simultaneously.

The CVRAT can be considered an extension of the CWRAT. The CVRAT only involves solving its test questions, that is, semantic association, whereas the CWRAT involves an additional feature, namely semantic association and compound word formation. According to the dual coding theory, people process verbal and visual information based on two independent and parallel systems that organize information *via* different connections (Paivio, 1971, 1986). Therefore, individuals can access target words through semantic association when answering both verbal and visual RAT questions, but the ways in which internal information is organized and connected during the two processes may differ because of their distinct stimuli. In other words, verbal and visual RATs examine different (verbal and visual) remote associative abilities.

At present, visual remote associates tests have been compiled in Slavic and Finnish (Toivainen et al., 2019). Nonetheless, these tests may not apply to the Chinese language because they are developed based on semantic associations. In this case, a picture may be interpreted differently in diverse cultural contexts. Furthermore, the involved semantic association may differ even though it conveys the same meaning in different cultures. Consequently, for the purposes of this research, the CVRAT was compiled after picture naming information was collected. It is expected that future researchers will provide another creativity measurement tool and complete the Chinese Remote Associates Test system.

### Creativity and Chinese Remote Associates Tests

Creativity refers to the ability to connect elements to establish new relationships to meet special needs (Mednick, 1962). The more people can associate seemingly irrelevant elements, the more creative they will be (i.e., remote associative ability). Mednick (1968) developed an RAT that boasts a short test duration and objective scoring to evaluate one’s remote associative ability. It has been proven effective in measuring creative potential and has been translated into multiple languages (Wu et al., 2020).

In Chinese-speaking countries and regions, Jen et al. (2004) referred to the semantic association employed by Mednick (1968) and took the initiative to apply it to CRAT, an RAT that conforms to the Chinese language. For instance, a CRAT question consisting of the Chinese characters “今” (chin; now), “輕” (ching; light), and “去” (chu; go), has the possible answer “年” (nien, year). Subsequently, another two verbal CRATs were completed, including the CCRAT, which required respondents to form compounds using Chinese two-character words (Huang et al., 2012), and the CRRAT, which asked respondents to form Chinese characters using Chinese character radicals (Chang et al., 2016). For instance, in a CCRAT question comprising stimuli words “市場” (shih-chang; market), “結束” (chieh-shu; an end), and “夕陽” (hsi-yang; sunset), one possible solution is “黃昏” (huang-hun; dusk) because the three stimuli are all associated with it. In a typical CRRAT, a question comprising the Chinese radicals “女” (nü; female), “子” (tzu; son), and “禾” (ho; standing grain), can be combined with “乃” (nai; be) to create three new Chinese characters as a possible solution. To summarize, existing CRATs have been compiled at the three levels of the Chinese language: Chinese characters, Chinese words, and Chinese radicals.

These CRATs can be used to measure creativity and may in fact test creativity in different dimensions because test questions, and the associated verbal knowledge and strategies, differ. Each of the three CRATs has two versions; hence, there are a total of six tests. It has been found that high-frequency target words and low-frequency stimuli lower the overall difficulty of test questions, while a growing number of associations can lead to an increasing degree of difficulty; this indicates that the same test question component in a CRAT question affects its difficulty (Hung and Wu, 2021). Regarding the external validity of CRATs, Wu (2019) analyzed the correlation of the performance on the three CRATs with verbal and visual divergent thinking tasks and insight problem-solving. Wu found that the CCRAT measures divergent thinking and remote association, the CWRAT can be used to assess insight problem-solving abilities, and the CRRAT is effective in evaluating remote visual association and insight problem solving. The above findings suggest a difference in the psychological attributes of the three CRATs evaluated.

Overall, CRATs of different kinds are compiled in the same way; the test questions, consisting of three stimuli, require respondents to propose a target word. In other words, respondents are asked to create new relationships based on independent elements (i.e., remote associations). However, the different language levels that CRATs involve may lead to distinct mechanisms of remote association (Wu, 2019). Moreover, it has been proved that typical RAT performance is significantly affected by verbal knowledge (Worthen and Clark, 1971). Pictures are another way for humankind to code information (Paivio, 1971, 1986). Therefore, developing an RAT using pictures to approach one’s visual remote associative mechanism is necessary. It also provides another perspective on CRAT development.

### Visual Remote Associates Tests and Its Properties

Replacing the verbal stimuli of a CRAT question with pictures is regarded as eliminating verbal and cultural influences (Olteteanu and Falomir, 2015). A VRAT question comprises three stimuli pictures, wherein respondents are asked to identify the meaning of a picture and propose a concept related to all three pictures simultaneously. For instance, in a VRAT question consisting of the pictures of door handles, gloves, and pens, the target word is “hand,” because door handles can be turned by a hand, gloves can be worn on a hand, and pens can be held by a hand (Toivainen et al., 2019; Olteţeanu and Zunjani, 2020). Overall, the cognitive process of VRAT involves picture recognition, semantic comprehension, and conceptual association. First, the individual must identify the pictures and evaluate whether they recognize these stimuli. Second, the individual must comprehend the meaning of each picture and its extended function or metaphor. Finally, based on the semantics of the three stimulus pictures, the individual must find another new concept that can connect functionally or meaningfully the three stimulus concepts simultaneously. The individual repeatedly thinks of possible concepts from the stimulus pictures, and cross-references them with each other’s stimulus concepts, and then may see the correct answer.

Like the CWRAT, the CVRAT emphasizes the semantic association between stimuli and target words. Hence, respondents are not dependent on their understanding of abstract words. Nevertheless, pictures visualize their stimuli and make them concrete, providing respondents with another way of coding information. Furthermore, the CWRAT has two problem-solving paths: “semantic association” and “compound word,” while the VRAT only focuses on “semantic association.” With verbal RATs as criterion-related tasks, previous research has found a low positive correlation between visual and verbal RATs (*r* = 0.30; Olteţeanu and Zunjani, 2020), revealing that the remote association they evaluate is not precisely the same. In this regard, it is necessary to compile a visual RAT for creativity assessment.

Nevertheless, empirical studies have not proved that VRATs can reduce the impact of different languages and cultures (Toivainen et al., 2019). Toivainen compared the performance of native Russian and Finnish speakers on the same VRAT and found that Finnish respondents had a higher average score than Russians. In addition, the correlation between their performance on verbal and visual RATs was analyzed. It was found that the performance of Russian speakers on the verbal RAT had a moderate positive correlation with their performance on the visual RAT, whereas the correlation was low for Finnish participants. This means that the native language that participants speak and the corresponding culture may affect their performances on verbal and visual RATs, respectively. This may occur because language users in different cultural contexts may make associations in distinct ways, which cannot be avoided by replacing verbal stimuli with visual ones. Furthermore, the compilation of the CCRAT has proved that test question components (such as stimuli and target words) affect the difficulty of an RAT and the evaluation of one’s remote associative ability, to which existing VRATs have not yet paid sufficient attention.

In addition to the association of stimuli with targets, whether stimuli pictures can be recognized effectively is another important topic for VRAT development, that is, picture naming. Picture naming involves a seemingly simple but in fact complicated cognitive process as a means of nonverbal communication for human beings (Pompéia et al., 2001). Its accuracy is influenced by multiple common factors, such as word frequency and name agreement. Word frequency has a cross-linguistic positive impact on naming performance (Laiacona et al., 2001; Bates et al., 2003; Kave, 2005). In other words, the higher the word frequency, the more likely it is for respondents to correctly name pictures. On the contrary, the lower the word frequency, the more likely they are to commit errors.

Name agreement refers to the extent to which respondents agree with a particular name for a specific concept (Snodgrass and Vanderwart, 1980). It exerts an impact on two successive processes: object recognition and lexical selection/phonological encoding. Moreover, it significantly influences the efficiency of contrasting, recalling, and recognizing pictures during the latency period (Cheng et al., 2010). Therefore, naming performance is affected by various pictorial factors. A standardized database is conducive to effectively using pictures as stimuli. At present, there is a database that consists of Snodgrass and Vanderwart pictures for children (ages four to six) and standardized data for name agreement of each picture, but it lacks adult samples (Wang et al., 2019). In this regard, it is necessary to establish a picture naming database that facilitates compiling CVRAT questions and thus developing a CVRAT.

In brief, it is of great significance and value to develop a CVRAT that applies to Chinese native speakers, with their culture and language taken into consideration, and is compiled based on a picture naming database that includes Chinese adult samples.

### The Present Study

Multiple CRATs have been developed, but they all use verbal stimuli; other materials have not yet been used as stimuli (Wu et al., 2020). In recent years, VRATs have been evaluated (Olteteanu and Falomir, 2015; Toivainen et al., 2019; Olteţeanu and Zunjani, 2020) and corresponding empirical studies have pointed out that cross-cultural differences still exist when verbal stimuli are replaced with visual stimuli (Toivainen et al., 2019). In this context, the present study aims to develop a CVRAT dedicated to traditional Chinese native speakers based on previous CRATs (Huang et al., 2012; Wu et al., 2017; Wu and Chen, 2017), with the aim of providing a new CRAT that can serve as a tool to assess remote association from a new perspective.

As an extension of the CWRAT, the CVRAT uses pictorial stimuli instead of verbal stimuli, emphasizing the semantic association between concepts. CVRAT questions require respondents to propose target words that are associated with all three visual stimuli. This study refers to how current VRATs are compiled (Olteteanu and Falomir, 2015; Toivainen et al., 2019; Olteţeanu and Zunjani, 2020) when developing a CVRAT. It includes the establishment of a database comprising high-name agreement pictures, on which CVRAT questions were compiled (with their reliability and validity) and were subsequently analyzed. To summarize, this study developed a CVRAT based on a standardized picture naming database, which applies to native Mandarin Chinese speakers. It is anticipated that different types of CRATs will enhance our understanding of RATs and complement the CRAT assessment system.

## Materials and Methods

### Stimulus Selection and Picture Naming

The CVRAT was compiled by referring to the development of the CWRAT (Huang et al., 2012). The stimuli and target words of a CVRAT question were selected from Chinese word association norms. The difference between a CVRAT and a CWRAT lies in the stimuli that they employ. Specifically, a CVRAT uses visual stimuli, whereas a CWRAT uses verbal stimuli. When a CVRAT is compiled, it should be guaranteed that respondents can clearly understand pictorial representations. Therefore, a collection of pictures with valid naming was established before the compilation.

Target and stimuli words were selected from the Chinese two-character word association norms and the associated word list (Hu et al., 2017). The concepts the words conveyed were represented in pictures. The pictures were used for subsequent CVRAT compilation, and the concept that each picture conveyed constituted the core of a CVRAT question. The word association norms provide information on word frequency, mental imagery, particularity, and commonality for each word. Word frequency is the number of a specific word’s occurrences among all words. Mental imagery is how well concepts form clear, mental, sensory experiences. Particularity is the degree of specificity of the associative response content. The higher the particularity, the more inconsistent the associative responses of the participants. Commonality is the degree of similarity in the content of associative responses. The higher the commonality, the more similar the associative responses of the participants. This study used the above indicators as criteria for word selection.

First, referring to the word selection rules of CWRAT (Huang et al., 2012), 95 two-character Chinese words were selected as the targets of CVRAT questions. We chose based word frequency, mental imagery (forming specific mental image representations), particularity (response words occurring alone, as a proportion of all associative responses), and commonality (total occurrences of the three most frequent words, as a proportion of all associative responses). Subsequently, three to five words were selected from the associated word list for each target word (i.e., a total of 350 Chinese words), after which pictures represented them as the stimulus pool of the CVRAT. To enable CVRAT questions to have different degrees of difficulty, this study set the word frequency between 0.01 and 1 time per 10,000 words (high word frequency), set mental imagery greater than 4.5 (so that respondents could access target words through associations based on pictures), set average commonality lower than 0.3, and average particularity less than 0.3 (to leave room for targets to be associated, to ensure they would not be accessed too easily). Stimuli came from the responses associated with the target words. Only those that could be represented by pictures and appeared in the database four times and above (excluding words associated the most frequently) were selected.

Second, stimuli were selected for each target based on the following criteria:

1. Stimuli should be semantically or functionally associated with the target. For instance, possible stimuli for the target word “喜帖” (hsi-tieh, wedding invitation) include “炸彈” (zha-dan, bomb), “結婚” (jie-hun, getting married), and “請客” (qing-ke, banquet), because “喜帖” (hsi-tieh, wedding invitation) is also called a red bomb, which is distributed when people get married to provide detailed information about wedding banquets.
2. Selected stimuli should not be associated semantically. In other words, the semantics of each stimulus word were not similar or closed. For example, the Chinese words “流汗” (liu-han, sweating), “悶熱” (men-je, sultry), and “季節” (chi-chieh, season) only lead respondents to a few target words like “夏天” (hsia-tien, summer). Still, their meanings are too close to each other conceptually, enabling respondents to access the target easily.
3. Selected stimuli should not correspond to multiple target words, as this would make it difficult for a test question to have a limited number of answers.

Subsequently, corresponding pictures of the 350 stimuli came from websites such as Pexels, Unsplash, Pixabay, Magdeleinem, PicJumbo, Burst, Flickr, Getty Images, and Visual Hunt. Three researchers were invited to judge whether the pictures corresponded to the stimuli. If two out of the three researchers believed that they did not match, the corresponding picture was selected and evaluated again. In this way, a total of 350 pictures matching the 350 stimuli were set.

Third, 350 pictures were randomly divided into three groups. There were approximately 120 pictures in each group, which were evaluated by 100 respondents. All pictures were randomly displayed on a screen for a maximum period of 20 s. Respondents pressed a button to enter the question-answer interface, and once they knew the picture’s name, they keyed in their response. They were granted 10 min to complete 20 pictures (i.e., 20 pictures per round), and they were given a three-minute break every two rounds. The entire process lasted approximately 60 min, with the instruction and breaks included. In addition, a picture naming task was conducted in each group. The researcher explained the purpose and schedule to respondents, who did not begin the task until they had signed the informed consent form after the explanation. When the experiment ended, they were rewarded with an NTD of $200.

Finally, the response items for each picture and the times at which the most frequent answers appeared were computed. The ratio of the most frequent answers to the total responses represents the name agreement of a picture. It was found that the average name agreement for the 350 pictures reached 0.92 with a standard deviation (SD) of 0.11, and those with name agreement of 0.75 and above were selected as visual stimuli.

### Items Development

**Figure 1:**
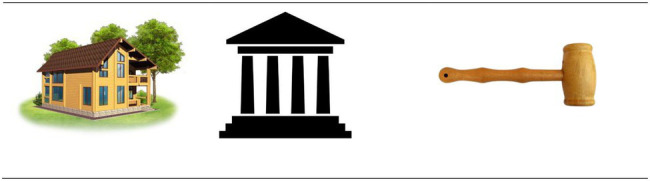
An example of a Chinese visual remote associates test item.

Based on the 95 selected target words and naming of stimuli pictures, CVRAT questions were compiled. At least three stimuli pictures needed to be identified for a target word to compile a CVRAT question. If a target word had fewer than three corresponding stimuli pictures (name agreement greater than 0.75), a test question with the target word as the answer could not be compiled. If five stimuli pictures had a name agreement of more than 0.75, two CVRAT questions were compiled. Using this method, 92 CVRAT questions were obtained, of which two served as examples, and the remaining 90 constituted questions to be answered by respondents. The 90 CVRAT questions were randomly divided into three CVRAT versions, each with 30 questions. To ensure that respondents had sufficient time to finish all test questions, the pilot versions allowed them 20 min for completion. Figure 1 is an example of the CVRAT question, which consists of three stimuli pictures that represent “房子” (fang-tzu, house), “法院” (fa-yuan, courthouse), and “錘子” (chui-tzu, hammer) respectively. A correct answer could be “拍賣” (pai-mai, auction), because the three pictures are all associated with it (i.e., a house is auctioned, items are auctioned in a courthouse, and a hammer is used during the auction).

### Pilot Study

#### Participants

A total of 268 college students participated in the pilot study. Because of the difference between the expected and actual numbers of different groups, respondents (87, 102, and 76) completed pilot versions of the CVRAT, respectively. Among them, 82 were male, with an average age of 20.77 (*SD* = 1.68). All participants were native traditional Chinese speakers with normal vision after correction. The Institutional Review Board approved the pilot study of the National Taiwan Normal University before it was conducted. The experiment did not begin until all participants fully understood it and signed an informed consent form.

The pilot study was conducted in groups, with each group taking the same version of the test. The experimenter explained the purpose and schedule of the experiment to the participants and asked them to sign the informed consent form. The answering time for the CVRAT is 20 min. Including informed consent, instructions, and testing, the total time is about 30 min. The procedures for the three versions of CVRAT are identical.

#### Formal Item Selection

Based on the results of the pilot studies, this study retained the test questions that had a moderate degree of difficulty (i.e., between 0.20 and 0.80) and a significant correlation with different CVRAT versions in terms of the total score (*r* > 0.25). Moreover, we screened out those with multiple different answers. After final selection, 40 test questions were retained to create two CVRAT versions, with 20 questions each. These questions were used for subsequent formal studies and the corresponding reliability and validity examinations.

## Formal Study

### Participants

In total, 450 adults participated in formal research, of which 170 were male and 280 were female. They ranged in age between 18 and 30 years old, with an average age of 20.98 (*SD* = 1.56). All participants were native Chinese Mandarin speakers with normal vision after correction. CVRAT A and CVRAT B were completed by 225 respondents each. They began the experiment after they had understood the research and signed an informed consent form.

### Criterion Tasks

The present study used the following tests as criterion tasks: CWRAT, CRRAT, CCRAT, insight problem-solving, and the divergent thinking test. Each task is described in detail below.

#### Chinese Word Remote Associates Test

The CWRAT used in this study was compiled by Huang et al. (2012). It consisted of 20 questions, each comprising three Chinese stimuli words. Respondents were asked to propose a Chinese word associated with all stimuli. For instance, a CWRAT question composed of “牛頓” (niu-tun; Newton), “蠟” (la; wax), and “紅色” (hung-se; red) would have “蘋果” (ping-kuo; apple) as a possible solution. Respondents scored one point for each correct answer, and the higher the score, the better their remote association. This test had two versions: CWRAT A and CWRAT B.

In terms of reliability and validity, the Cronbach’s *α* was 0.81. With insight problem-solving as the criterion task, it was found that CWRAT had a positive correlation with insight problem-solving (*r* = 0.51). The New Creativity Test was adopted to examine discriminant validity, which revealed that it did not correlate with any of the indicators (*r* between −0.06 and 0.08). In summary, the CWRAT had good validity and reliability.

#### Insight Problem

Compiled by Chiu (2005, Unpublished)^1^, the insight problem-solving test used in this study had six questions. Among them, there were three figural questions and three verbal questions. To avoid errors caused by prior knowledge of the questions, respondents were asked whether they had come across the question and knew its answer before the experiment. If respondents had known the answer, their response was set as “missing,” and the score was excluded from their total score. As for the scoring, respondents scored one point for each correct answer and zero for each incorrect answer. The sum of the points obtained for each question constituted their total scores. Therefore, the highest possible score was 6, while the lowest was 0.

As for its validity and reliability, Chiu (2005, Unpublished) (see footnote 1) recruited 125 college students as research participants. The internal consistency coefficient *α* was 0.52, which is acceptable according to the standard proposed by Kline (1998). As for validity, confirmatory factor analysis was conducted, which found that it conformed to the standard of the overall fitness (*χ*^2^(124) = 7.72, *p* > 0.05, GFI = 0.98, SRMR = 0.048, PNFI = 0.51, CFI = 1.00). This suggests that insight problems involve a potential construct. Regarding its structural fitness, all *λ* parameters reached the level of significance and the composite reliability of the factors was 0.51, indicating that the insight problem test reached the standard of internal fitness. The internal consistency coefficient was 0.50 based on the scores of these 236 participants in this study.

#### Chinese Radical Remote Associates Test

The CRRAT used in this study was compiled by Chang et al. (2016). It consisted of 20 questions, each comprising three Chinese radicals as stimuli, and required respondents to propose one that could be paired with them to make three commonly seen legitimate Chinese characters. For instance, if a CRRAT question is composed of the stimuli “女” (nü; female), “子” (tzu; son), and “禾” (ho; standing grain), one possible answer is “乃” (nai; be). Respondents were given one point for each correct answer, and the higher the score, the better the ability to form a remote association.

As for its reliability and validity, its Cronbach’s *α* coefficient was 0.70, and it had a positive correlation with insight problem-solving (*r* = 0.42), as shown by criterion-related evidence. Moreover, the New Creativity Test was employed to analyze its discriminant validity, finding that it did not correlate with divergent thinking indicators (*r* ranged between −0.10 and 0.15). Therefore, it had good reliability and validity.

#### Chinese Compound Remote Associates Test

The CCRAT used in this study was compiled by Wu et al. (2017). It consisted of 20 questions, with each question comprising three stimuli of Chinese characters. Respondents were asked to think of a Chinese character combined with all three stimuli to create three meaningful two-character Chinese words. For example, if a CCRAT question is comprised of “療” (liao; treatment), “防” (fang; defense), and “統” (tung; completely), the Chinese character “治” (chih, rule) can be paired with them to form the words “治療” (chih-liao; treatment), “防治” (fang-chih; prevention), and “統治” (tung-chih; ruling), respectively. Participants scored one point for each correct answer, and the higher the score, the better the remote associative ability. In addition, their performance was represented by the pass rate (i.e., the percentage of correct answers).

Its reliability was analyzed based on the Cronbach’s *α* coefficient, which was 0.69 (Hung and Wu, 2021). The respondents’ performance on CCRAT was positively correlated with verbal and figural divergent thinking (rs = 0.27, 0.23) and insight problem-solving (*r* = 0.12). Therefore, this test had considerable reliability and validity.

#### Divergent Thinking Test

Developed by Wu et al. (1998), the divergent thinking test used in this study consists of two versions: verbal and figural. This research only employed the verbal one as the criterion task, asking respondents to propose unusual uses for bamboo chopsticks that are typically used to have meals and pick up food. Respondents were scored in terms of fluency, flexibility, and originality after completing the test.

Regarding reliability, Kendall’s coefficient of concordance was computed using the performance of 20 respondents who scored multiple points. The coefficient was employed for inter-rater reliability, and the corresponding results were as follows: fluency (*r* = 0.96), flexibility (*r* = 0.97), and originality (*r* = 0.94). With the Torrance Creative Thinking Test (TTCT) as the criterion task, it was found that a majority of indicators had significant correlations: fluency (0.52–0.75), flexibility (0.47–0.62), originality (0.09–0.57), and elaboration (0.39).

### Procedure

The present research conducted the CVRAT by referring to how the CWRAT was performed (Huang et al., 2012). Respondents were asked to propose legitimate two- or three-character Chinese words that are not proper names in a specific field, such as the name of a person, place, or movie. However, it was recommended to limit to respond using only two-character Chinese word to maintain the consistency of the number of characters in the answer for future use. The experiment was conducted across all groups. In total, 225 respondents filled out CVRATs A and B, which took about 10 min. They were required to complete the test in a limited time period, and their concentration levels were also assessed.

Moreover, participants completed three of the following five criteria tasks because too long response time may affect their concentration: a CWRAT, insight problem-solving, CRRAT, CCRAT, or the divergent thinking test. They were asked to complete each of the criterion tasks in approximately 10 min. The entire experiment lasted approximately 60 min, which included the instructions and breaks between the tests. After the experiment, participants were rewarded with NTD $200 worth of gift cards.

### Data Analysis

The obtained data were analyzed as follows: first, the degree of difficulty for each question and that of each version were computed; Cronbach’s *α* was then computed as the internal consistency reliability value; finally, the following correlations were computed as the criterion-related validity value of the CVRAT: the overall pass rate (i.e., percentage of correct answers) for CVRATs A and B, CWRAT, CRRAT, CCRAT, insight problem-solving, and the correlations between fluency, flexibility, and originality of the divergent thinking test (Table 1).

**Table 1:** Correlations between Chinese visual remote associates test and criterion tasks.

	CRAT			Divergent thinking test		
				IP			
	CCRAT	CRRAT	CWRAT		Fluency	Flexibility	Originality
CVRAT A	0.21^*^	0.41^**^	0.31^**^	0.29^**^	0.08	0.15	0.05
CVRAT B	0.23^*^	0.26^*^	0.49^**^	0.37^**^	−0.09	−0.02	−0.19^*^

* *p* < 0.05;

** *p* < 0.01.

CVRAT, Chinese visual remote associates test; CRAT, Chinese remote associates test; CCRAT, Chinese compound remote associates test; CRRAT, Chinese radical remote associates test; CWRAT, Chinese word remote associates test; IP, insight problem.

### Reliability

This study analyzed the internal consistency reliability of CVRATs A and B using data from the formal study. CVRATs A and B had Cronbach’s *α* coefficients of 0.52 and 0.53, respectively, consistent with insight problem-solving (Chiu, 2005, Unpublished; see footnote 1). Thus, its reliability was acceptable.

In addition, CVRATs A and B did not show significant differences in the degree of difficulty (*t* (448) = 0.10, *p* = 0.924, *d* = 0.01). Their overall degrees of difficulty were 0.48 (*SD* = 0.15) and 0.48 (*SD* = 0.15), respectively, indicating that CVRAT A can function as a duplicate of CVRAT B, and vice versa.

### Validity

The study used CCRAT, CRRAT, CWRAT, insight problem-solving, and divergent thinking tests as the criterion tasks. CVRAT A was found to have a significantly positive correlation with CCRAT (*r* = 0.21, *p* = 0.032, *n* = 108), CRRAT (*r* = 0.41, *p* < 0.001, *n* = 80), CWRAT (*r* = 0.31, *p* = 0.001, *n* = 110), and insight problem-solving (*r* = 0.29, *p* = 0.002, *n* = 111). Nevertheless, there was no significant correlation between CVRAT A and the fluency, flexibility, and originality of the divergent thinking test (rs = 0.08, 0.15, 0.05, ps = 0.334, 0.063, 0.526, *n* = 149).

Moreover, CVRAT B was positively correlated with CCRAT (*r* = 0.23, *p* = 0.011, *n* = 120), CRRAT (*r* = 0.26, *p* = 0.025, *n* = 76), CWRAT (*r* = 0.49, *p* < 0.001, *n* = 136), and insight problem-solving (*r* = 0.37, *p* < 0.001, *n* = 125). Nonetheless, it was not significantly correlated with fluency (*r* = −0.09, *p* = 0.322, *n* = 113) and flexibility (*r* = −0.02, *p* = 0.804, *n* = 113) in the divergent thinking test and had a poor negative correlation with originality (*r* = −0.19, *p* = 0.044, *n* = 113).

## General Discussion

This study took the initiative to use pictures with Chinese naming information as stimuli to compile a CVRAT that applies to native speakers of Chinese Mandarin, which constitutes a tool that evaluates one’s conceptual association. The research findings showed that the CVRAT, whose reliability was acceptable, had good criterion-related validity with the CWRAT, CRRAT, CCRAT, and insight problem-solving. Moreover, it had appropriate discriminant validity with fluency, flexibility, and originality in divergent thinking. In other words, the CVRAT can effectively evaluate remote associative ability as a tool that measures one’s figural creativity.

In terms of reliability, the two (formal) versions of the CVRAT had internal consistency reliability coefficients of 0.52 and 0.53 respectively, indicating that respondents’ performance on CVRAT questions lacked stability. In other words, the constructs that the CVRAT measures did not have high homogeneity. This may have resulted from the different ways in which respondents interpret stimuli pictures. Pictures with high name agreement were chosen to compile the test questions, but it is inevitable that respondents may have interpreted them differently. The interpretation of a picture is closely related to whether respondents can solve problems smoothly, which affects the consistency of their performance on each question. Low internal reliability may also consequentially affect the validity of assessing remote association *via* CVRAT. Therefore, future research might examine different item dimensions according to the collected data and only select some items. Simultaneously, the CWART data could be collected. In this regard, subsequent research can investigate the cognitive factors involved in each test question (Hung and Wu, 2021), which would facilitate compiling a CVRAT that measures construct more consistently.

Regarding validity, CWRAT, CRRAT, CCRAT, insight problem-solving, and the divergent thinking test were used to examine the criterion-related and discriminant validity of the CVRAT. First, the CVRAT was significantly correlated with the three verbal CRATs, namely the CWRAT, CRRAT, and CCRAT, indicating that they may involve the same attribute (i.e., remote association). However, the correlations between the RATs were different, suggesting that the CVRAT shares other common constructs with the three verbal CRATs.

This research referred to the CWRAT when compiling the CVRAT questions. The CWRAT was positively correlated with the CVRAT. The difference between the two lies in the stimuli; the former used verbal stimuli, while the latter employed visual stimuli. The positive correlation between the two indicates that both can be utilized to evaluate one’s conceptual associative ability. Moreover, the CWRAT also involves the way Chinese words are combined (Huang et al., 2012). As a result, the two tests showed only a moderate correlation.

In addition, the CRRAT had the second strongest positive correlation with the CVRAT. Among the three verbal CRATs, the CRRAT has a relatively high positive correlation with the CWRAT, and it is also deemed applicable to the assessment of one’s insight problem-solving (Chang et al., 2016; Wu, 2019). Thus, the positive correlation between the CRRAT and the CVRAT reflects that they may both involve insight problem-solving.

Moreover, there was only a low positive correlation between the CCRAT and CVRAT. Unlike the other two verbal CRATs, the CCRAT focuses on a more remote association; thus, it has a significant positive correlation with divergent thinking but a low correlation with insight problem-solving (Wu et al., 2017; Wu, 2019). In other words, the CCRAT has different measurement attributes from the CWRAT and CRRAT. In summary, the correlations between the CVRAT and the three verbal CRATs conform to expectations, i.e., the CVRAT is like the CWRAT in terms of measurement attributes; they both measure one’s conceptual association. However, CVRAT respondents accessed a concept through pictures, whereas CWRAT participants accessed it *via* words.

The CVRAT also had a moderately positive correlation with insight problem-solving. As mentioned above, the CVRAT shares similar measurement attributes with the CWRAT, which is positively correlated with insight problem-solving (Huang et al., 2012). Thus, the positive correlation between CVRAT and insight problem solving indirectly supports the notion that the CVRAT and CWRAT share the same measurement attribute, which echoes the known research finding that RATs and insight problem-solving share a similar process (Bowden and Jung-Beeman, 2003).

Nonetheless, the CVRAT had no significant positive correlation with the fluency, flexibility, and originality of the verbal divergent thinking test. As seen from the structure of test questions, RATs and divergent thinking tasks are opposites; RATs involve a close-ended problem-solving process, while divergent thinking tasks are open-ended (Wakefield, 1992). Therefore, existing studies employ divergent thinking tasks to analyze the discriminant validity of the CWRAT and CRRAT (Huang et al., 2012; Chang et al., 2016). Similar to previous findings, this study found that the CVRAT and the divergent thinking test measure different constructs. In addition, this study did not use the figural divergent thinking test as the criterion as it requires individuals to add lines based on a specific shape (i.e., “人”), then form other figures, such as a pen or a house (Wu et al., 1998). It differs from the CVRAT’s focus on recognizing images and connecting concepts.

This study had some limitations. First, CVRATs A and B do not have high internal consistency reliability. This is also the case with the insight problem (Chiu, 2005, Unpublished; see footnote 1). This problem may be common to this type of test. In this regard, subsequent research may analyze the internal components of each test question and compile a version with higher internal consistency. In addition, the stimuli and targets of CVRAT questions mainly originated from Chinese two-character word norms (Hu et al., 2017). Normative data include 400 high-frequency and high-imagery words, but not all associated responses can be visualized, leading to a limited number of stimuli being used for test compilation. At the same time, we did not control for part-of-speech of the target word, which may also have affected the quality of the measures. Notably, the current version of CVRAT was developed based on Traditional Chinese users in Taiwan, hence it may not be suitable for other types of Chinese users. Lastly, despite the use of non-verbal stimuli in the CVRAT, whether CVRAT performance does not directly correlate with verbal intelligence remains to be verified.

Overall, the CVRAT developed in this study provides a new remote association measurement tool for Chinese language users. It could be used to understand the individual’s semantic association capability. At the same time, CVRAT expands the types of stimuli of the RAT and initially realizes the possibility of various kinds of remote associations. In the future, research can continue to explore more creativity-related topics based on the CVRAT. For instance, subsequent research can examine whether individuals experience different problem-solving processes when undertaking verbal and non-verbal RATs. It can also compare the performance of participants of different genders and ages on the CVRAT and their understanding of the pictures of the test questions. In addition, it can approach the difference in function in terms of different groups of CVRAT respondents (such as gender, culture, and generations).

## Data Availability Statement

The raw data supporting the conclusions of this article will be made available by the authors, without undue reservation.

## Ethics Statement

The studies involving human participants were reviewed and approved by Institutional Review Board of National Taiwan Normal University. The patients/participants provided their written informed consent to participate in this study.

## Author Contributions

C-LW collected and analyzed the data and wrote the initial draft of the manuscript. P-ZC collected the data and assisted in literature review. H-CC designed this study. All authors approved the final version of the paper.

## Funding

This work was financially supported by the “Institute for Research Excellence in Learning Sciences” of National Taiwan Normal University (NTNU) from The Featured Areas Research Center Program within the framework of the Higher Education Sprout Project by the Ministry of Education (MOE) in Taiwan. The authors also thank the Ministry of Science and Technology, Taiwan, R.O.C, for funding this study, through projects on “How do individuals display their creativity in groups? The moderation effects of intrinsic and extrinsic factors, brain structure and interactive mobile teaching” (MOST109-2628-H-003-005-MY2).

## Conflict of Interest

The authors declare that the research was conducted in the absence of any commercial or financial relationships that could be construed as a potential conflict of interest.

## Publisher’s Note

All claims expressed in this article are solely those of the authors and do not necessarily represent those of their affiliated organizations, or those of the publisher, the editors and the reviewers. Any product that may be evaluated in this article, or claim that may be made by its manufacturer, is not guaranteed or endorsed by the publisher.

## Footnotes

^1^Chiu, F. C. (2005). The Cognitive Process of Creativity. Unpublished doctoral dissertation. Taipei, Taiwan: National Taiwan Normal University.
